# Efficacy of SPI-1865, a novel gamma-secretase modulator, in multiple rodent models

**DOI:** 10.1186/alzrt173

**Published:** 2013-04-18

**Authors:** Robyn M Loureiro, Jo Ann Dumin, Timothy D McKee, Wesley F Austin, Nathan O Fuller, Jed L Hubbs, Ruichao Shen, Jeff Jonker, Jeff Ives, Brian S Bronk, Barbara Tate

**Affiliations:** 1 Satori Pharmaceuticals, 281 Albany St., Cambridge, MA 02139, USA

## Abstract

**Introduction:**

Modulation of the gamma-secretase enzyme, which reduces the production of the amyloidogenic Aβ_42 _peptide while sparing the production of other Aβ species, is a promising therapeutic approach for the treatment of Alzheimer's disease. Satori has identified a unique class of small molecule gamma-secretase modulators (GSMs) capable of decreasing Aβ_42 _levels in cellular and rodent model systems. The compound class exhibits potency in the nM range *in vitro *and is selective for lowering Aβ_42 _and Aβ_38 _while sparing Aβ_40 _and total Aβ levels. *In vivo*, a compound from the series, SPI-1865, demonstrates similar pharmacology in wild-type CD1 mice, Tg2576 mice and Sprague Dawley rats.

**Methods:**

Animals were orally administered either a single dose of SPI-1865 or dosed for multiple days. Aβ levels were measured using a sensitive plate-based ELISA system (MSD) and brain and plasma exposure of drug were assessed by LC/MS/MS.

**Results:**

In wild-type mice using either dosing regimen, brain Aβ_42 _and Aβ_38 _levels were decreased upon treatment with SPI-1865 and little to no statistically meaningful effect on Aβ_40 _was observed, reflecting the changes observed *in vitro*. In rats, brain Aβ levels were examined and similar to the mouse studies, brain Aβ_42 _and Aβ_38 _were lowered. Comparable changes were also observed in the Tg2576 mice, where Aβ levels were measured in brain as well as plasma and CSF.

**Conclusions:**

Taken together, these data indicate that SPI-1865 is orally bioavailable, brain penetrant, and effective at lowering Aβ_42 _in a dose responsive manner. With this unique profile, the class of compounds represented by SPI-1865 may be a promising new therapy for Alzheimer's disease.

## Introduction

Alzheimer's disease (AD) is a severe neurodegenerative disease that is defined by two pathological features, amyloid plaques and neurofibrillary tangles. Because amyloid plaques appear before the onset of clinically-defined dementia symptoms, neurodegeneration and subsequent cognitive impairment are hypothesized to be a downstream consequence of β-amyloid (Aβ) peptide dysregulation [1-3]. Aβ peptides are small fragments cleaved from a much larger integral membrane protein, the amyloid precursor protein (APP). In the AD cascade, APP is cleaved initially by β-secretase (BACE), leaving the C99 fragment in the membrane, which is then cleaved by gamma-secretase, an aspartyl protease complex [4,5]. Gamma-secretase continues to make sequential cleavages every three to four amino acids [6-9], resulting in Aβ fragments ranging in size from 49 to fewer than 34 amino acids [10,11]. Much of the focus in AD research has been on Aβ_42_, since it has been shown to be the most amyloidogenic and neurotoxic fragment [12-14]. More recently, Aβ_43 _has also been shown to have these detrimental properties [15]. To test the hypothesis that lowering Aβ_42 _levels may slow the progression of or prevent AD, multiple amyloid-targeted therapeutic approaches have been developed and moved into human clinical trials. These include Aβ clearance-directed immunotherapies as well as inhibitors of BACE or gamma-secretase enzyme activities, both of which are required for Aβ production.

To prevent the production of these neurotoxic Aβ peptides, researchers have focused on developing small molecule inhibitors of BACE and gammasecretase. In preclinical animal models, *in vivo *administration of gamma secretase inhibitors led to severe side effects, including an increased number of goblet cells in the intestine and decreased intrathymic differentiation and lymphocyte development [16-18]. These adverse events were found to be the result of inhibiting gamma-secretase's ability to process other substrates, specifically NOTCH [19-21], which is critical for cell development and differentiation [22]. Similar adverse events were also observed in recent clinical trials of semagacestat and avagacestat, further suggesting that complete inhibition of gamma-secretase is not a viable approach [23-25]. Much remains unknown about the approach to prevent Aβ production through BACE inhibition, as a subset of those molecules are currently in human clinical trials [26].

The discovery of multiple structural classes of compounds that modulate gamma-secretase activity, instead of inhibiting it, offers the potential promise of avoiding the mechanism-based adverse events observed with gamma-secretase inhibitors. Gamma-secretase modulators (GSMs) are observed to decrease the production of the more amyloidogenic Aβ_42 _peptide, while preserving total Aβ levels and sparing gamma-secretase cleavage of the other substrates, such as NOTCH. Modulation allows the initial cleavage of substrates, but alters the processivity of the enzyme by shifting the production of Aβ peptides to the shorter, non-amyloidgenic forms without affecting the total level [27]. A first generation GSM, Flurizan from Myraid Genetics was tested in a Phase 3 trial. However, the compound is a weak modulator (IC_50 _= 250 μM), lacks brain penetration, and produced side effects [10,28]. The compound failed to show efficacy, and development was halted in 2008 [29]. Since the approach of gamma modulation has not been adequately tested in humans, it is still believed that a more potent, drug-like compound could be a viable therapeutic approach.

**Figure 1:**
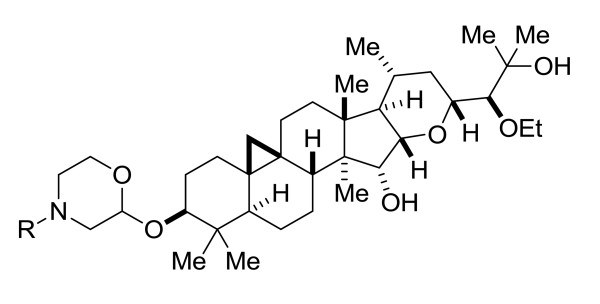
**General structure of Satori gamma-secretase modulators (GSMs), including SPI-1865**.

Here we describe the *in vivo *pharmacology for a novel series of compounds, represented by SPI-1865 (Figure 1). All compounds within the series that have been tested *in vivo *show a PK/PD relationship in rodents as robust as SPI-1865 (data not shown). The compound scaffold is derived from an initial hit, a triterpene glycoside (SPI-014), which was discovered through a screen of a natural products library for compounds with GSM properties [30]. Following a comprehensive and focused research effort, compounds like SPI-1865 were identified [31,32]. The compounds in this series, including SPI-1865, have a novel and proprietary structure, as well as the unique effect on the Aβ profile of lowering both Aβ_42 _and Aβ_38_, without effecting Aβ_40 _in cellular systems [33]. Using immunoprecipitation/mass spectrometry (IP/MS) analysis of conditioned media from Satori compound-treated versus control 2B7 cells, it was observed that the total Aβ levels are maintained with concomitant lowering of Aβ_38 _and Aβ_42 _and increases in Aβ_37 _and Aβ_39 _[34]. Furthermore, since increasing substrate levels do not result in an IC_50 _shift, it is likely that SPI-1865 binds to the gamma-secretase complex as do other GSMs [35-37] instead of the APP substrate [33,34]. In the studies described here, the effects of SPI-1865 on Aβ_38_, Aβ_40 _and Aβ_42 _in both wild-type and transgenic animals were examined. The Aβ changes observed in these studies reflect the changes observed in our cellular systems, resulting in a decrease of both Aβ_38 _and Aβ_42 _in all three models, suggesting that SPI-1865 maintains its GSM properties *in vivo*.

## Materials and methods

### Test compound

SPI-1865 was prepared in a manner as described in Bronk *et al*., patent WO2011109657 A1 20110909. Merck GSM1 was prepared as described in Madin *et al*., patent WO2007116228 A120071018.

### Cell culture and compound treatment

CHO-2B7 cells (Mayo Clinic) are Chinese hamster ovary cells stably transfected with human βAPP 695wt [38,39]. The cells were cultured in Ham's F12 media (Thermo Fisher SH30026.01, Waltham, MA, USA) supplemented with 10% FBS, 0.25 mg/mL Zeocin and 90 ug/mL penicillin/streptomycin at 37°C in a 5% CO_2 _atmosphere. For compound treatment, cells were plated in 96-well plates at a density of 1.0 × 10^5 ^cells/mL and allowed grow to 100% confluence over two days. Test compounds in dimethyl sulfoxide (DMSO) were diluted 100-fold directly into the media before being adding to the cells. Immediately prior to adding compound-containing media to the cells, the cells were washed once with 1X PBS. Conditioned media from CHO-2B7 cells were collected after 5 hrs of treatment and the levels of Aβ peptides were assessed as described below.

### Aβ *in vitro *assay measurement

Conditioned media were collected after 5 hrs of treatment and diluted with one volume of MSD blocking buffer (1% BSA in MSD wash buffer). MSD Human (6E10) Aβ 3-Plex plates, which are pre-spotted with three separate spots in each well containing capture antibodies against the unique C-terminal ends of Aβ_38_, Aβ_40 _and Aβ_42_, respectively, were blocked with MSD blocking buffer for one hour. Samples were transferred to the blocked plates with 6E10 detection antibody and incubated for 2 hrs at room temperature with orbital shaking followed by washing and reading according to the manufacturer's instructions (SECTOR^® ^Imager 2400 Meso Scale Discovery, Gaithersburg MD, USA).

### *In vivo *study methods

The animal handling and procedures were performed either at Agilux Laboratories in Worcester, MA, USA, or Cerebricon in Kuopio, Finland. All animal handling and procedures were conducted in full compliance to AAALAC International and NIH regulations and guidelines regarding animal care and welfare. These protocols were reviewed and approved by Agilux's or Cerebricon's respective Institutional Animal Care and Use Committees (IACUC) prior to any activities involving animals.

Female transgenic mice (Tg2576, 3 months of age; *n *= 20), wild-type male CD-1 mice (six weeks of age; *n *= 8 to 12) or wild-type male Sprague Dawley rats (200 to 225 g body weight; *n *= 12) were utilized to assess *in vivo *efficacy.

For wild-type rat and mouse studies, all animals were acclimated to the test facility for a minimum of two days prior to initiation of the study. Compounds were dosed orally in 10:20:70 ethanol/solutol/water or 10:20:70 ethanol/cremaphor/water via oral gavage. Samples were harvested at either at 6 or 24 hrs post dose for Aβ and compound exposure measurements. Blood samples were collected into K_2_ethylenediaminetetraacetic acid (EDTA) and stored on wet ice until processed to plasma by centrifugation (3,500 rpm at 5°C) within 30 minutes of collection. Each brain was dissected into three parts: left and right hemispheres and cerebellum. Brain tissues were rinsed with ice cold PBS (without Mg^2+ ^or Ca^2+^), blotted dry and weighed. Plasma and cerebella were analyzed for parent drug via liquid chromatography/tandem mass spectrometry (LC/MS/MS). Parent drug levels were compared to a standard curve to establish the plasma and brain levels.

In the transgenic studies, compound was dosed in 10:20:70 ethanol/solutol/water via oral gavage. Samples were harvested at 24 hrs post dose for Aβ and compound exposure measurement. The mice were subjected to cisterna magna puncture and collection of cerebrospinal fluid (CSF) (approximately 5 μl per mouse). Individual CSF samples were flash-frozen on dry ice and stored at -80°C. Thereafter, the mice were subjected to cardiac puncture and blood samples were collected into K_2_EDTA tubes and stored on wet ice until processed to plasma by centrifugation (2,000 g at 4°C for 10 minutes) within 30 minutes of collection. The plasma was aliquoted for both Aβ measurements and parent drug using LC/MS/MS. Both sets of tubes were frozen at -80°C. The brains were perfused with non-heparinized saline (the blood flushed away) and removed carefully. Brains were rinsed with ice cold PBS (without Mg^2+ ^or Ca^2+^), blotted dry, dissected on ice into three pieces (left and right hemisphere and cerebellum). Samples were frozen in liquid nitrogen immediately prior to storage at -80°C. Cerebella were analyzed for parent drug via LC/MS/MS. Parent drug levels were compared to a standard curve to establish the plasma, brain and CSF levels.

### Rodent Aβ determination

This protocol is a modification of protocols described by Lanz and Schachter [40] and Rogers *et al. *[41,42]. Frozen hemispheres were weighed into tared homogenization tubes (MP Biomedicals#6933050 for rat; MP Biomedicals, Solon, OH, USA) and (Simport#T501-4AT; Simport, Beloeil, Qc, Canada) containing one 5-mm stainless steel bead (Qiagen#69989 for mouse). For every gram of brain, 10 mLs of either 6 M guanidine hydrochloride (wild-type rat and mouse) or 0.2% diethyl amine in 50 mM NaCl (transgenic mouse) was added to the brain-containing tubes on wet ice. Rat hemispheres were homogenized for one minute and mouse hemispheres were homogenized for 30 seconds at the 6.5 setting using the FastPrep-24 Tissue and Cell homogenizer (MP Biomedicals#116004500). Homogenates were rocked for 2 hrs at 4°C, then pre-cleared by ultracentrifugation at 100,000 × *g *for one hour at 4°C. Pre-cleared wild-type rat and mouse homogenates were concentrated over solid phase extraction (SPE) columns (Oasis HLB 96-well SPE plate 30 μm, Waters#WAT058951; Waters Corp., Milford, MA, USA). Briefly, SPE columns were prepared by wetting with 1 mL of 100% methanol followed by dH_2_0 using vacuum to pull liquids through. Brain homogenates were then added to the prepared columns (1.0 mL from rat and 0.7 mL from wild-type mouse). Columns were washed twice with 1 mL of 10% methanol followed by two 1 mL washes with 30% methanol. Labeled eluent collection tubes (Costar cluster tubes #4413; Corning Inc., Corning, NY, USA) were placed under SPE columns and samples were eluted under very mild vacuum with 300 μL of 2% NH_4_OH/90% methanol. Eluents were dried to films under vacuum with no heat in a speed vacuum microcentrifuge. Films were resuspended in 150 μL of Meso Scale Discovery (MSD, Gaithersburg, MD, USA) blocking buffer (1% BSA in MSD wash buffer) for one hour at room temperature with occasional vortexing. Transgenic mouse plasma (50 μL) was extracted in 500 μL of 6 M guanidine hydrochloride briefly at room temperature and then 450 μL was concentrated over SPE columns and dried to films as described above. Transgenic plasma films were resuspended in 225 μL of MSD blocking buffer. Pre-cleared transgenic mouse brain homogenates were diluted and neutralized as follows. A volume of 45 μL of pre-cleared transgenic mouse brain homogenates were diluted into 450 μL of blocking buffer and were neutralized with 5 μL of 0.5 M Tris pH 6.8. For Aβ_38_, Aβ_40 _and Aβ_42 _measurements, MSD 96-well multi-spot Human/Rodent (4G8) Aβ triplex ultra-sensitive ELISA plates, which are pre-spotted with three separate spots in each well containing capture antibodies against the unique C-terminal ends of Aβ_38_, Aβ_40 _and Aβ_42_, respectively, were blocked with MSD blocking buffer for one hour at room temperature with orbital shaking. A volume of 25 μL of neat resuspended wild-type rat or mouse brain homogenates were added in duplicates to the blocked 3-plex Aβ MSD plates with SULFO-TAG 4G8 antibody (MSD). Diluted and neutralized transgenic mouse brain homogenates, neat resuspended transgenic plasma samples or transgenic mouse CSF samples (diluted 1:10 in MSD blocking buffer) were added as described above to blocked MSD 96-well multi-spot Aβ triplex ultra-sensitive ELISA plates with SULFO-TAG 6E10 antibody (MSD). The Aβ 3-Plex plates were incubated for 2 hrs at room temperature with orbital shaking followed by washing and reading according to the manufacturer's instructions (SECTOR^® ^Imager 2400, MSD). The average Aβ concentrations from duplicate measurements of each animal were converted to percent vehicle values and the treatment group averages were statistically compared by analysis of variance (ANOVA). Statistical significance was defined as *P *< 0.01 in all experiments.

## Results

### SPI-1865 decreases Aβ_42 _and Aβ_38 _in 2B7 cells

**Figure 2:**
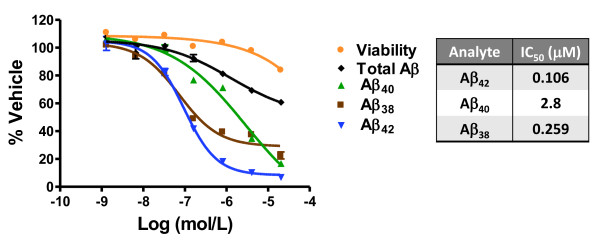
***In vitro *pharmacology of SPI-1865, a unique gamma-secretase modulator that lowers β-amyloid (Aβ)_42 _and Aβ_38_**. CHO-2B7 cells were treated with increasing concentrations of SPI-1865. Conditioned media were collected after 5 hrs of treatment and the levels of Aβ peptides were assessed. The values for each Aβ_38_, Aβ_40 _and Aβ_42 _for each of the doses are expressed as a percentage of the Aβ levels from vehicle-treated cells. IC_50_'s of Aβ_38 _(259 nM), Aβ_42 _(106 nM) and Aβ_40 _(2.8 μM) were calculated by using the inflection point or extrapolated from the curve at Aβ_xx _50% lowering, as appropriate. The listed values were averaged from multiple experiments and a representative dose response is shown here.

CHO-2B7 cells, which over-express human wild-type APP, were treated with increasing concentrations of SPI-1865. Conditioned media from CHO-2B7 cells were collected after 5 hrs of treatment and the levels of Aβ peptides were assessed using the MSD 3-Plex assay for Aβ_42_, Aβ_40 _and Aβ_38_. As shown in Figure 2, SPI-1865 reduces both Aβ_38 _and Aβ_42 _with an IC_50 _of 259 nM and 106 nM, respectively. Aβ_40 _was found to have an IC_50 _of 2.8 μM, resulting in > 20-fold selectivity for Aβ_42 _over Aβ_40_. Total Aβ only decreased at doses where cytotoxicity was observed, indicating that SPI-1865 is capable of modulating gamma-secretase processivity, not inhibiting enzyme activity.

### SPI-1865 reduces Aβ_42 _and Aβ_38 _with a single oral dose in Sprague Dawley rats

**Figure 3:**
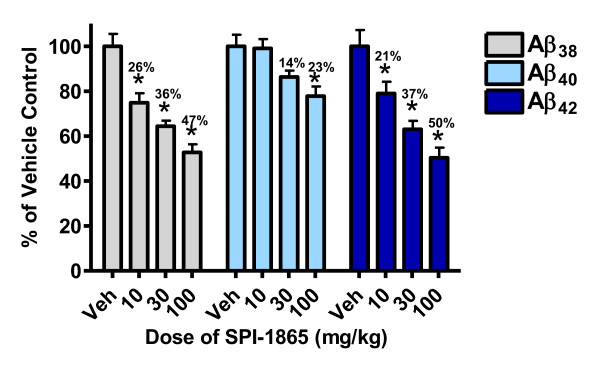
**SPI-1865 dose-responsively lowers β-amyloid (Aβ)_42 _and Aβ_38 _after a single oral dose**. Sprague Dawley rats were orally administered a single dose of SPI-1865 of 10, 30 or 100 mg/kg. Plasma and brain were harvested 24 hrs post dose and analyzed for compound, Aβ_38_, Aβ_40_, and Aβ_42 _levels. Data are graphed as a percent of vehicle control. *P-value < 0.01 based on analysis of variance (Dunnett's test).

The compound was assessed for efficacy in Sprague Dawley rats. SPI-1865 has a delayed Tmax and half-life in excess of 24 hrs following a single oral dose in rats (Table 1). Based on this profile, compound efficacy was examined using a single oral dose and tissues were harvested 24 hrs post dose. Male Sprague Dawley rats were administered an oral dose of 10, 30 or 100 mg/kg SPI-1865 in a formulation of 10/20/70 of ethanol/cremaphor/water. The average amounts of Aβ_38_, Aβ_40 _and Aβ_42 _(pg Aβ/g of brain) for the vehicle-treated group of rats were 312 ± 21, 3,094 ± 192 and 682 ± 61, respectively. As shown in Figure 3, at all doses, a significant lowering of brain Aβ_42 _and Aβ_38 _levels was observed compared to vehicle-treated animals. Aβ_40 _was significantly reduced at only the highest dose by 22 ± 5% (average percent lowering ± standard error of the mean, SEM). The decreases in Aβ_42 _levels were dose-responsive and correlated with the exposures in both brain and plasma. In the rats dosed with 10 mg/kg SPI-1865, brain levels reached 2.8 ± 0.3 μM and plasma levels were 3.3 ± 0.1 μM, which resulted in a lowering of Aβ_42 _by 21 ± 6% relative to vehicle control. The compound levels increased with the 30 mg/kg dose to 11 ± 1 μM in the brain and 8.5 ± 0.3 μM in the plasma and resulted in a 37 ± 5% decrease in Aβ_42_. In the 100 mg/kg dose group, brain levels reach 33 ± 2 μM and plasma levels were 14 ± 1 μM of SPI-1865, leading to an Aβ_42 _reduction of 50 ± 5%. Similar changes occurred with brain Aβ_38 _levels, resulting in dose-responsive reductions of 26 ± 5, 36 ± 3 and 47% ± 5 upon the administration of 10, 30 and 100 mg/kg SPI-1865, in that order. These data demonstrate that SPI-1865 is capable of modulating gamma-secretase *in vivo *and results in a similar Aβ profile as observed *in vitro*.

**Table 1:** Pharmacokinetic properties of SPI-1865 in mouse and rat

Species	Volume (L/kg)^2^	Brain/plasma (24 h)^1^	T_1/2 _(h)^2^	Tmax
Mouse	9.2	0.4 to 1.4	8.3	approximately 4 hrs
Rat	5.8	0.5 to 1.5	129	6 to 8 hrs

The pharmacokinetic properties of SPI-1865 were assessed in both mouse and rat. ^1^Based on various oral dosing regimens; ^2^Volume and T_1/2 _values are based on intravenous drug delivery data. T_1/2_, half-life; Tmax, time of maximum concentration.

### Efficacy in Sprague Dawley rats is improved following multiple day dosing

**Figure 4:**
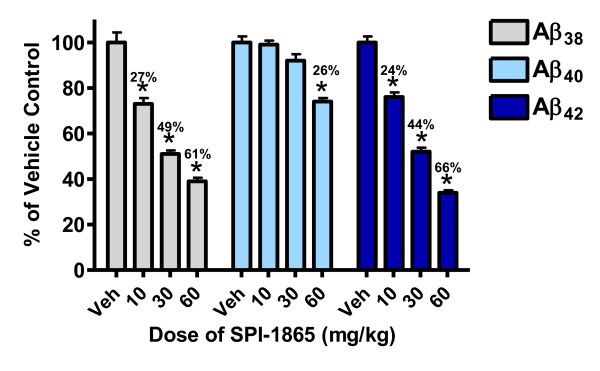
**SPI-1865 dose-responsively lowers β-amyloid (Aβ)_42 _and Aβ_38 _after multiple oral doses**. Sprague Dawley rats were orally administered SPI-1865 for six days, once a day (QD){AU query: ok as defined? yes} at a dose of 10, 30 or 60 mg/kg. Plasma and brain were harvested 24 hrs post the final dose and analyzed for compound, Aβ_38_, Aβ_40_, and Aβ_42 _levels. Data are graphed as a percent of vehicle control. *P-value < 0.01 based on analysis of variance (Dunnett's test).

To further investigate the effects of SPI-1865 in the rat, a multiple-day study was performed. Male Sprague Dawley rats were orally dosed once a day for six days with 10, 30 or 60 mg/kg of SPI-1865. The average amounts of Aβ_38_, Aβ_40 _and Aβ_42 _(pg Aβ/g of brain) for the vehicle-treated group of rats were 98 ± 47, 2,690 ± 92 and 840 ± 32, respectively. As shown in Figure 4, the 10 mg/kg dose resulted in brain levels of 4.4 ± 0.2 μM, plasma levels of 8.0 ± 0.4 μM, and approximately a 25% reduction in both brain Aβ_38 _and Aβ_42 _levels with no significant alteration in Aβ_40 _as compared to vehicle control. The 30 mg/kg dose lowered both Aβ_38 _and Aβ_42 _levels in the brain by roughly 44%, with brain exposures of 16 ± 1 μM and plasma levels 13 ± 1 μM, once again without significantly affecting Aβ_40_. At the highest dose, Aβ_42 _was reduced by 66 ± 1% at exposures of approximately 45 μM (brain) and 19 ± 1 μM (plasma). At this highest dose, Aβ_40 _was significantly lowered by 26 ± 2%. For all doses, Aβ_38 _levels were lowered to a similar degree as Aβ_42_. This demonstrates that multiple-day administration of SPI-1865 can result in higher compound exposures and enhanced lowering of Aβ_42 _levels.

### SPI-1865 reduces Aβ_42 _and Aβ_38 _in both brain and plasma of Tg2576 mice

The use of transgenic mouse models, which over-express human APP, allows for the measurement of Aβ peptides in three compartments: the brain, plasma and CSF. In this study, three month old female Tg2576 mice (*n *= 20 per group) were administered SPI-1865 for six days, which is sufficient to reach steady state exposures based on previous murine pharmacokinetic analysis (data not shown). The mice received 10, 30, 60 or 90 mg/kg of SPI-1865 orally once a day for six days or a positive control, Merck GSM-1, orally once on day six. Samples were harvested 24 hrs post dose for SPI-1865 and 6 hrs post dose for GSM-1. CSF and blood were directly harvested and the brain was perfused prior to collection.

**Figure 5:**
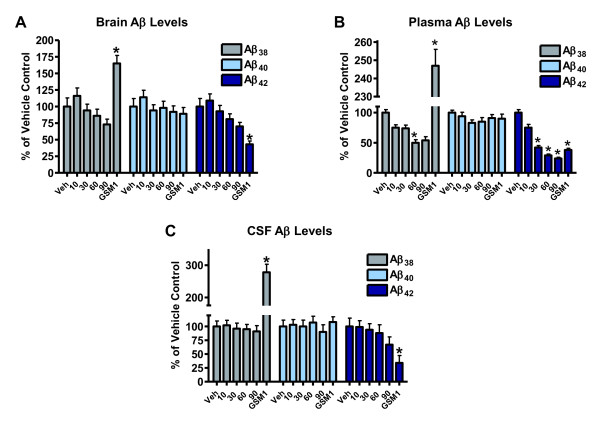
**Efficacy data for SPI-1865 in human amyloid precursor protein (APP)-overexpressing transgenic mice**. SPI-1865 was orally dosed once a day (QD) for six days in female Tg2576 mice. Tissues were harvested 24 hrs after the final dose. The levels of compound, β-amyloid (Aβ)_38_, Aβ_40_, Aβ_42 _were measured in brain and plasma. Aβ_38_, Aβ_40_, Aβ_42 _levels were also measured in cerebrospinal fluid (CSF). All values are graphed as % of vehicle control. *P-value < 0.01 based on analysis of variance (Dunnett's test).

As shown in Figure 5, dose-responsive decreases in Aβ_38 _and Aβ_42 _were observed in plasma with SPI-1865. In the brain, there was a trend towards lowering Aβ_38 _and Aβ_42 _levels, but the changes did not reach statistical significance. Aβ_42 _seemed to be somewhat lower in the 90 mg/kg dose group in the CSF, but the change was also statistically insignificant. The average amounts of Aβ_38_, Aβ_40 _and Aβ_42 _in the brain (pg Aβ/g of brain), for the vehicle-treated group of Tg mice were 4,202 ± 547, 44,052 ± 5,262 and 6,470 ± 812, respectively. In the plasma of the vehicle-treated Tg2576 mice, average Aβ_38_, Aβ_40 _and Aβ_42 _levels were found to be 412 ± 21 6,266 ± 251 and 1,294 ± 66 pg Aβ/g and 4,384 ± 446, 35,137 ± 4,111 and 5,057 ± 785 pg Aβ/g in the CSF, respectively. At the highest dose, an Aβ_42 _reduction of 76 ± 2% in the plasma was observed, with a plasma exposure of 6.4 ± 0.5 μM as measured 24 hrs post the final dose. The 10 mg/kg-treated group had no significant effect on any brain Aβ levels with drug levels in brain of 0.5 μM and plasma drug levels of 1 μM. The 30 and 60 mg/kg treatment groups had more pronounced reductions, which correlated with the increased brain and plasma exposures. For example, in the 60 mg/kg group, compound levels reached 5.4 ± 0.5 μM in the plasma and 3.9 ± 0.4 μM in the brain that resulted in a decrease in Aβ_38 _by 50 ± 2.4% and Aβ_42 _by 71 ± 2% in the plasma, respectively. Merck GSM-1 significantly reduced Aβ_42 _and increased Aβ_38 _levels. In this study, large variability was observed in both the brain and CSF samples, possibly due to blood contamination in both tissues. The variability was also observed in the vehicle and positive control groups, indicating that this is not a consequence of SPI-1865 treatment.

Most interestingly, when examining the total data set from the transgenic mouse study, the plasma reductions stand out as being significantly greater than those in brain and CSF. It has been reported that the Tg2576 mice do produce peripheral Aβ and SPI-1865 could be impacting those sources directly [43]. Other studies with SPI-1865 indicate that the compound is highly protein bound, with 97.6% bound in the plasma and approximately 99.9% in the brain. When we calculate the free fraction in plasma from the 90 mg/kg dose group where there was 76% lowering of plasma Aβ_42_, the free plasma fraction is calculated to be 154 nM which exceeds the IC_50 _of 106 ± 19 nM. If we perform a similar comparison focusing on plasma levels using the 30 mg/kg group, where 58% lowering was observed, the free plasma levels are found to be 60 nM, which with inherent assay variability is reasonably close to the *in vitro *IC_50 _of 106 nM. These data suggest that free plasma concentrations of SPI-1865 are driving the efficacy that was observed in the periphery. In the brain, the highest dose resulted in a non-significant 30% lowering and a calculated brain free fraction of 6.9 ± 6 nM, which is in the range of the IC_25 _of 33 ± 18 nM. With the difficulty in measuring brain protein binding accurately when the free fraction is quite small, the high variability in measuring Tg2576 brain Aβ_42 _*in vivo *and the fact that there were no significant data points for brain Aβ_42 _lowering, we needed another study investigating free brain versus free plasma concentrations as the cause of Aβ_42 _reduction in the central compartment.

### SPI-1865 lowers Aβ_42 _and Aβ_38 _in wild-type CD-1 mice

**Figure 6:**
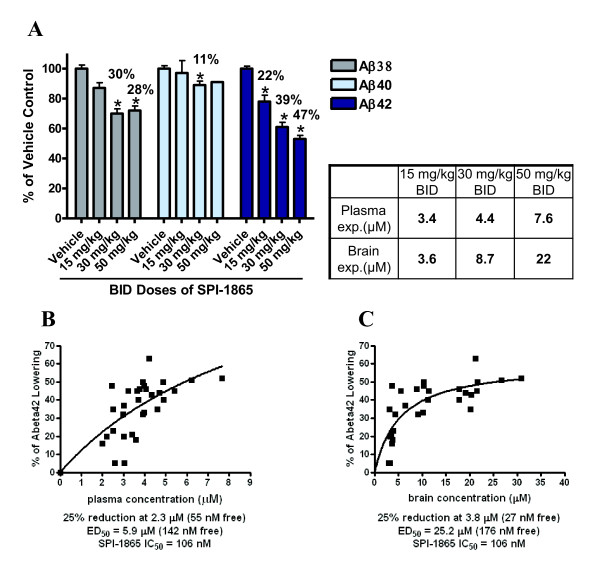
**Steady-state efficacy of SPI-1865 in wild-type mice**. SPI-1865 was orally dosed twice daily (BID) for six days in male CD-1 mice. Tissues were harvested 6 hrs after the final dose. The levels of compound, β-amyloid (Aβ)_38_, Aβ_40_, Aβ_42 _were measured in brain. Compound levels were also measured in plasma. (**A**) Aβ levels are graphed as % of vehicle control. *P-value < 0.01 based on analysis of variance (Dunnett's test). The percent Aβ_42 _lowering for each of the animals in this study was then plotted against the concentrations of SPI-1865 in the plasma (**B**) and brain (**C**). A best-fit line was plotted using GraphPad and the ED50s were extrapolated and shown under each graph with the IC50 of SPI-1865 in cells.

To further examine the effects of SPI-1865, an improved study design in wild-type mice, which have much less variability in Aβ levels, was performed. However, due to very low plasma levels of Aβ in wild-type mice, we are unable to measure efficacy in plasma. Based on the half-life of 8 hrs and a Tmax of approximately 4 hrs in the mouse, samples were collected 6 hrs post dose to optimize the measurement of Aβ and compound exposures. A twice-daily (BID) dosing regimen for six days was selected to ensure steady state was attained and maximize the time SPI-1865 was engaged with the enzyme complex. The results of this treatment are shown in Figure 6. The average amounts of Aβ_38_, Aβ_40 _and Aβ_42 _in the brain (pg Aβ/g of brain) for the vehicle-treated group of wild-type mice were 324 ± 8.5, 4,465 ± 98 and 1,012 ± 18, respectively. A significant lowering of brain Aβ_42 _was observed at all doses. Brain Aβ_38 _was also significantly lowered at the two top doses and a slight, but significant lowering of 11% was observed for brain Aβ_40 _levels at the 30 mg/kg dose. The total compound exposures in the 50 mg/kg BID group were found to be 7.6 ± 0.3 μM in the plasma and 22 ± 1 μM in the brain where brain Aβ_42 _was decreased by 47 ± 2%. The 30 mg/kg BID treatment reduced brain Aβ_42 _by 39 ± 2% with a total plasma exposure of 4.4 ± 0.3 μM and brain levels of 8.7 ± 1 μM. In the 15 mg/kg BID-treated animals, brain exposures of 3.6 ± 0.1 μM and a plasma exposure of 3.4 ± 0.2 μM to reduce brain Aβ_42 _by 22%.

The percent Aβ_42 _lowering of each dosed animal was plotted against the plasma (Figure 6B) and brain exposures (Figure 6C) in μM. Best-fit lines were superimposed over the data revealing the roughly hyperbolic nature of the PK/PD relationship in BID-dosed mice. From these lines, the plasma and brain exposure levels required for 25% and 50% Aβ_42 _lowering were extrapolated. We found that 25% Aβ_42 _lowering correlated with a 2.3 μM exposure in the plasma and 3.8 μM exposure in the brain and the exposure levels associated with 50% Aβ_42 _lowering were 5.9 and 25.2 μM in the plasma and brain, respectively. We compared brsain free-fraction levels to the efficacy and once again found the exposures to be below the IC_25 _(33 ± 18 nM) even at the highest dose where 22 ± 1 nM was achieved and corresponded with 47% reduction. Taken together with the Tg2576 data, we cannot determine if brain free-fraction plays a role in SPI-1865 efficacy. Conversely, plasma free-fraction, and the observed efficacy in the brain, appear to be strongly correlated. At the highest dose, plasma free-fraction was calculated to 182 nM, which resulted in a 47% reduction in brain Aβ_42_, while the 30 mg/kg dose group was determined to have a plasma free-fraction of 105 nM (39% lowering of brain Aβ_42_) and the low dose, a plasma free-fraction of 82 nM (22% lowering of brain Aβ_42_). With an *in vitro *Aβ_42 _IC_50 _of 106 ± 19 nM and an IC_25 _of 33 ± 18 nM, there is a significant correlation between plasma free-fraction and brain efficacy. At all doses, the plasma free-fraction was slightly higher than the amount of compound expected to induce the same response *in vitro*, but the numbers are within the range of variability. Taken together these data further support the Tg2576 data that plasma free-fraction of SPI-1865 correlates with brain efficacy.

## Discussion

The studies described here demonstrate that SPI-1865 is a novel modulator of gamma-secretase, which is capable of lowering both Aβ_42 _and Aβ_38 _in multiple animal models. In Sprague-Dawley rats, the compound effectively lowered Aβ_42 _levels following a single dose. With once a day dosing for six days, the Aβ_42 _and Aβ_38 _response was improved. Given that the half-life of SPI-1865 is greater than 24 hrs in the rat, improved efficacy was anticipated, since accumulation of compound in the plasma occurs following multiple days of dosing. However, the compound levels in the brain did not show the same degree of accumulation with multiple dosing, even though brain efficacy was improved. Based on multiple *in vitro *studies (data not shown), SPI-1865 is not a substrate for transporters nor does it cause CYP induction, either of which may lower the brain exposure. Together these data suggest that sustained exposure to SPI-1865 levels over multiple days contributes to enhanced efficacy of the compound to modulate gamma-secretase.

SPI-1865 was next examined for efficacy in 3-month-old, female Tg2576 mice. These mice produce human APP with the SWE mutation via the prion promoter [44]. The transgene expression has been shown to be highest in the brain, but the transgene is also expressed in peripheral tissues [43]. The peripheral production of Aβ in the Tg2576 mouse may influence the effects of SPI-1865 on brain Aβ versus plasma Aβ levels when comparing efficacy in transgenic and wild-type models.

Data from this study indicate that SPI-1865 readily crosses the blood-brain barrier, as evident in the levels of SPI-1865 measured in brain. The ability to measure efficacy in both the brain and plasma compartments in the transgenic mice allows us to probe whether total or free concentrations of compound drives efficacy. If total compound were responsible for efficacy in both tissue compartments, one would expect to see similar decreases in Aβ_42 _and Aβ_38 _in both the plasma and brain in the Tg2576, as the exposure in each compartment is similar. However, what is observed is a significantly improved lowering of Aβ_42 _and Aβ_38 _in the plasma relative to the brain. The most likely reason for the difference in efficacy between the two compartments is the level of free drug available in the plasma versus the brain. The level of compound binding in the plasma is 97.6% based on *in vitro *studies, leaving 2.4% free compound to interact with gamma-secretase. This is a significantly higher free fraction than the estimates for free fraction in the brain, where protein binding was measured at approximately 99.9%. When the average free fraction for each compartment is compared for the 90 mg/kg dose (brain_total _= 6.9 μM; plasma_total _= 6.4 μM), the plasma free-fraction levels of 153 nM exceed the *in vitro *IC_50 _of SPI-1865 (106 ± 19 nM) and plasma Aβ_42 _levels were lowered by 76 ± 2%. The average brain free-fraction was only 6.9 nM, below the IC_25 _of 33 ± 18 nM, and while a trend toward lowering of Aβ_42 _was observed, it was not statistically significant_. _It is important to note the high variability of Aβ_42 _measurement in the Tg2576 brains impacted the ability to see statistically significant decreases in brain Aβ_42_, even with a large number of animals per group (*n *= 20) and may affect our determination of free versus bound in the brain. Moreover, it is challenging to get an accurate measure of free concentration in brain when brain protein binding is greater than 99%. Even in the face of these technical challenges, the data presented here indicate that the plasma free-fraction of SPI-1865 correlates most closely with Aβ lowering in that compartment.

In the Tg2576 study, the changes in CSF Aβ levels were examined along with the plasma and brain. While treatment with SPI-1865 trended towards a decrease in brain Aβ_38 _and Aβ_42 _and significantly lowered the plasma levels of Aβ_42 _at the three highest doses tested, in the CSF the Aβ peptide levels were not significantly decreased. There are several possible explanations for the lack of a significant effect. The high variability in CSF Aβ measurements within this study may mask an effect on the Aβ_38 _and Aβ_42 _levels. In addition, the timing of sample collection relative to dosing may have been more optimal to assess plasma changes than to assess changes in brain and CSF Aβ levels_._

In wild-type mice, we further investigated the *in vivo *activity of SPI-1865 once steady state plasma levels were achieved. With a half-life of 8 hrs and a Tmax of approximately 4 hrs in the mouse (Table 1), a six-day BID dosing study was designed to measure exposure and Aβ levels 6 hrs post dose, allowing maximal efficacy to be observed by capturing exposures near the maximal plasma concentration and providing nearly continuous engagement with the enzyme. This is different from the Tg2576 study where once-a-day dosing was utilized for six days and samples were harvested 24 hrs post dose. In this study, there was much less variability of Aβ levels among animals than the Tg2576 mice and six days of BID dosing resulted in a significant lowering of brain Aβ_42 _at all doses compared to vehicle-dosed animals. Plasma and CSF levels were not measured in this experiment. When we examined plasma free-fraction versus efficacy, an Aβ_42 _lowering of 22 ± 4%, 39 ± 3% and 47 ± 2% occurred as the doses increased, and these changes corresponded with free plasma concentrations of 82, 105 and 182 nM, respectively. Together, this wild-type mouse study in combination with the Tg2576 model and rat data, demonstrate the ability of SPI-1865 to lower both Aβ_38 _and Aβ_42 _*in vivo*.

## Conclusions

Taken together the data shown in these studies demonstrate that SPI-1865 is an efficacious gamma-modulator *in vivo *(all *in vivo *data are summarized in Table 2). This is demonstrated in multiple rodent models using a single dose, multiple-day dosing or a multiple-day BID dosing regimen. These data indicate that SPI-1865 is orally bioavailable, brain penetrant, and has a different Aβ profile from other GSMs, both *in vitro *and *in vivo*. SPI-1865 lowers Aβ_42 _and Aβ_38 _while sparing Aβ_40 _levels. This novel GSM pharmacology is dose-responsive, driven by the plasma free-fraction of the compound and is capable of reducing both Aβ_42 _and Aβ_38 _levels in APP over-expressing mice. Overall, SPI-1865 exemplifies the unique Aβ profile and good drug-like properties of SPI compounds, and further suggests they may be novel therapeutic approaches for Alzheimer's disease.

**Table 2:** Summary of SPI-1865 PK/PD studies in wild-type and transgenic mouse and wild-type rat

Species and dosing regime	Dose (mg/kg)	Tissue	Plasma exposure (μM)	Brain exposure (μM)	%Aβ38 lowering	%Aβ40 lowering	%Aβ42 lowering	Collection time (hrs)
Wild-type rat single dose	10	Brain	3.3 ± 0.1	2.8 ± 0.3	26 ± 5	1 ± 5	21 ± 6*	24
	30	Brain	8.5 ± 0.3	11 ± 1	36 ± 3*	14 ± 4	37 ± 5*	24
	100	Brain	14 ± 1	33 ± 2	47 ± 5*	22 ± 5*	50 ± 5*	24
								
Wild-type rat multiple doses	10	Brain	8.0 ± 0.4	4.4 ± 0.2	27 ± 3*	1 ± 2	24 ± 2*	24
	30	Brain	13 ± 1	16 ± 1	49 ± 2*	8 ± 3	44 ± 2*	24
	60	Brain	19 ± 1	45 ± 4	61 ± 2*	26 ± 2*	66 ± 1*	24
								
Tg2576 mouse multiple doses	10	Brain	1.1 ± 0.2	0.5 ± 0.1	-16 ± 12	-14 ± 11	-9 ± 10	24
	30	Brain	2.5 ± 0.1	1.3 ± 0.1	6 ± 9	6 ± 9	7 ± 9	24
	60	Brain	5.4 ± 0.5	3.9 ± 0.4	14 ± 10	2 ± 10	19 ± 8	24
	90	Brain	6.4 ± 0.5	6.9 ± 0.6	27 ± 8	8 ± 9	30 ± 6	24
	10	Plasma	1.1 ± 0.2	0.5 ± 0.1	25 ± 5	6 ± 7	25 ± 5*	24
	30	Plasma	2.5 ± 0.1	1.3 ± 0.1	26 ± 5	17 ± 5	58 ± 3*	24
	60	Plasma	5.4 ± 0.5	3.9 ± 0.4	50 ± 5*	15 ± 7	71 ± 2*	24
	90	Plasma	6.4 ± 0.5	6.9 ± 0.6	46 ± 6*	9 ± 6	76 ± 2*	24
	10	CSF	1.1 ± 0.2	0.5 ± 0.1	-2 ± 9	-3 ± 9	1 ± 11	24
	30	CSF	2.5 ± 0.1	1.3 ± 0.1	4 ± 10	0 ± 11	6 ± 11	24
	60	CSF	5.4 ± 0.5	3.9 ± 0.4	5 ± 9	-7 ± 12	22 ± 15	24
	90	CSF	6.4 ± 0.5	6.9 ± 0.6	9 ± 11	10 ± 13	33 ± 14	24
								
Wild-type mouse multiple twice-daily doses	15	Brain	3.4 ± 0.2	3.6 ± 0.1	13 ± 4	3 ± 2	22 ± 4*	6
	30	Brain	4.4 ± 0.3	8.7 ± 1.0	30 ± 3*	11 ± 2*	39 ± 3*	6
	50	Brain	7.6 ± 0.3	22 ± 1	28 ± 2*	9 ± 2	47 ± 2*	6

The pharmacokinetic and pharmacodynamic properties of SPI-1865 were assessed in mouse and rat model systems. *Statistically significant lowering of the indicated β-amyloid (Aβ) species compared to vehicle-treated animals (*P *< 0.01). CSF, cerebrospinal fluid. Values are given as the mean ± standard error of the mean.

## Abbreviations

Aβ: β-amyloid; AD: Alzheimer's disease; ANOVA: analysis of variance; APP: amyloid precursor protein; BACE: beta-secretase; BID: bis in diem or twice a day; BSA: bovine serum albumin; CSF: cerebrospinal fluid; DMSO: dimethyl sulfoxide; EDTA: ethylenediaminetetraacetic acid; ELISA: enzyme-linked immunosorbent assay; FBS: fetal bovine serum; GSM: gamma-secretase modulator; IP/MS: immunoprecipitation/mass spectrometry; LC/MS/MS: liquid chromatography/tandem mass spectrometry; QD: quaque die, or once a day; PBS: phosphate-buffered saline; SEM: standard error of the mean; SPE: solid phase extraction.

## Competing interests

All authors are, or have been employees with Satori Pharmaceuticals.

## Authors' contributions

RML carried out the processing and analysis of the study samples, aided in study design, and contributed to the drafting of the manuscript. JAD designed the studies and coordinated with the contract research organizations to ensure studies were performed as designed and contributed to the drafting of the manuscript. TDM developed the *in vitro *assay used for compound assessment and produced the *in vitro *data shown here and edited manuscript drafts. WFA, NOF, JLH, RS and BSB designed and produced the chemical matter utilized in the described studies. JJ and JI provided support and advice for these studies. BT oversaw the biology efforts, ensured data quality and edited manuscript drafts. All authors read and approved the final manuscript.
